# Challenges in AVNRT ablation in a patient with previous atrio-pulmonary Fontan surgery: a case report

**DOI:** 10.1093/ehjcr/ytaf569

**Published:** 2025-11-07

**Authors:** Avinash Jeewooth, Atul Kaushik, Aparna Jaswal, Amitesh Chakravarty

**Affiliations:** Department of Electrophysiology, Fortis Escorts Heart Institute (FEHI), New Delhi 110025, India; Department of Cardiology, Fortis Escorts Heart Institute, Okhla, New Delhi 110025, India; Department of Electrophysiology, Fortis Escorts Heart Institute (FEHI), New Delhi 110025, India; Department of Electrophysiology, Fortis Escorts Heart Institute (FEHI), New Delhi 110025, India

**Keywords:** Ablation, AVNRT, Case Report, Fontan surgery, SVT

## Abstract

**Background:**

Patients with complex congenital heart defects, such as tricuspid atresia, often require palliative surgical interventions like the Fontan procedure to optimize systemic and pulmonary circulation. While these surgeries improve survival rates, they are associated with long-term complications, including arrhythmias, due to significant anatomical and electrophysiological alterations. Among these, atrioventricular nodal reentrant tachycardia (AVNRT) is relatively uncommon but poses substantial challenges in diagnosis and management.

**Case Summary:**

This case report describes a 36-year-old male with a history of tricuspid atresia and atrio-pulmonary Fontan surgery who presented with symptomatic recurrent palpitations. Electrophysiological (EP) study revealed an atypical variant of AVNRT, necessitating a complex ablation strategy. The unique anatomical considerations in this patient, including a hypoplastic right ventricle, a dilated and tortuous coronary sinus, and difficulty in defining the Triangle of Koch, complicated the identification of the compact atrioventricular (AV) node. Conventional catheter placement techniques were inadequate, and a retrograde aortic approach was employed to localize His bundle potentials on the left side of the septum. Advanced 3D electroanatomic mapping was used to construct a virtual geometry of the right atrium, which guided the identification and ablation of the slow pathway. Despite challenges in re-inducing the tachycardia during the procedure, successful slow pathway modification was achieved, and no recurrence of tachycardia was observed during follow-up.

**Discussion:**

Fontan anatomy poses unique procedural risks and requires a multidisciplinary approach involving paediatric interventional cardiologists, radiologists, and electrophysiologists. The findings of this case underscore the critical importance of pre-procedural planning, detailed anatomical assessment, and advanced imaging techniques to achieve favourable outcomes.

Learning point

- Fontan anatomy with various congenital heart defects poses an increased risk of arrhythmias and due to structural and technical differences requires a thorough workup and planning for managing such arrhythmias with ablation techniques.
- A tailor-made approach for every such case is required with the indubitable use of advanced 3D mapping system during EPS and ablation.

## Introduction

Patients with complex congenital heart defects, such as tricuspid atresia, undergo staged palliative surgeries, including the Fontan procedure, to optimize circulation. While these surgical interventions improve survival, they also predispose patients to arrhythmias due to anatomical and haemodynamic alterations. AVNRT, although less common than intra-atrial reentrant tachycardia (IART) in this cohort, poses diagnostic and therapeutic challenges.

Fontan-associated anatomic variations, such as dilated atria, absent or hypoplastic ventricles, and altered positioning of the atrioventricular node, complicate conventional ablation strategies. In this case report, we discuss a patient with tricuspid atresia post-Fontan surgery who developed symptomatic AVNRT.

## Summary figure

**Figure:**
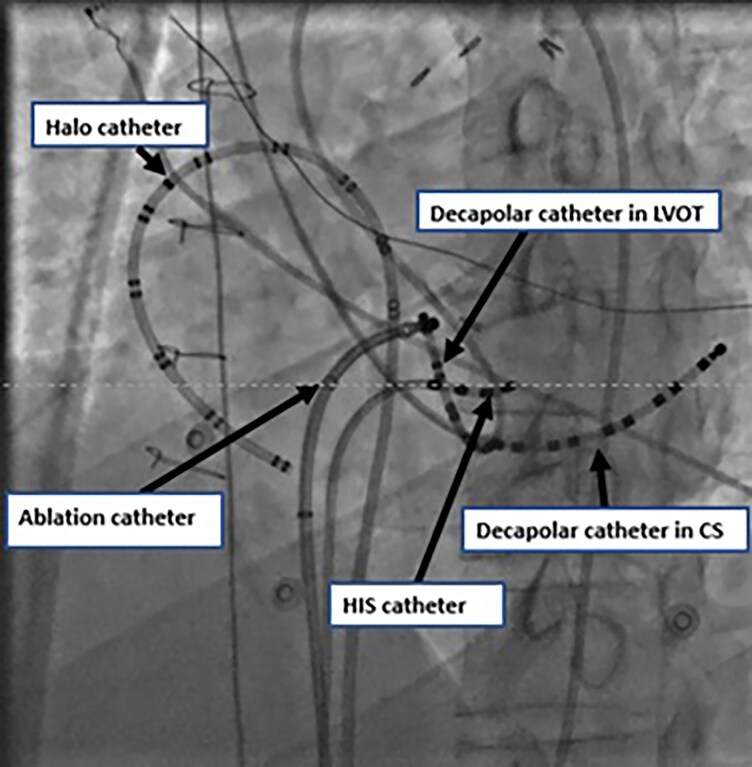


## Case presentation

A 36-year-old male patient presented with recurring episodes of palpitations. He underwent modified Blalock-Taussig shunt for tricuspid atresia at the age of 3years, followed by a classical Fontan procedure in 1999 and closing the atrial septal defect. Patient presented with BP of 110/60 mmhg, pulse rate 88/min, respiratory rate 19/min with NYHA class II.

He previously had a documented episode of narrow complex short-RP tachycardia. His baseline ECG showed sinus rhythm. He was on tab diltiazem 120 mg/day. Due to the recurrence of symptomatic tachycardia that was interfering with his daily life, he was scheduled for an EP study.

Transthoracic echocardiography (TTE) revealed a functioning Fontan circuit with tricuspid atresia and a hypoplastic RV with normal LV function. These findings were confirmed by a chest CT, and CT-coronary angiography (CAG) revealed normal coronaries (Supplementary material online, *Figure S1*). Right heart catheterization showed patent intra-cardiac Fontan circuit, dilated right atrium(RA), and patent superior vena cava (SVC) and Inferior vena cava (IVC) with normal flow. There were no other Fontan-related complications.

The EP study began with the insertion of a duo-decapolar (Halo) catheter via IVC, which was positioned in the right atrium. A decapolar catheter was then inserted via SVC with an attempt to cannulate the coronary sinus (CS). However, due to difficulty in locating the CS-ostium, levophase CAG was performed, revealing a dilated and tortuous CS (see Supplementary material online, *Figure S2*). The CS was successfully cannulated. A quadripolar catheter was inserted via the IVC to record His potentials. Locating the compact AV node proved challenging, so the decision was made to access the AV node from LV via a retrograde aortic approach. A decapolar catheter was placed in LVOT, successfully recording His and left bundle potential (see Supplementary material online, *Figure S3*). It served as backup pacing, as RV catheter could not be placed due to the hypoplastic right ventricle (RV). Additionally, a quadripolar catheter was used to localize the septal tricuspid annulus.

**Figure 1:**
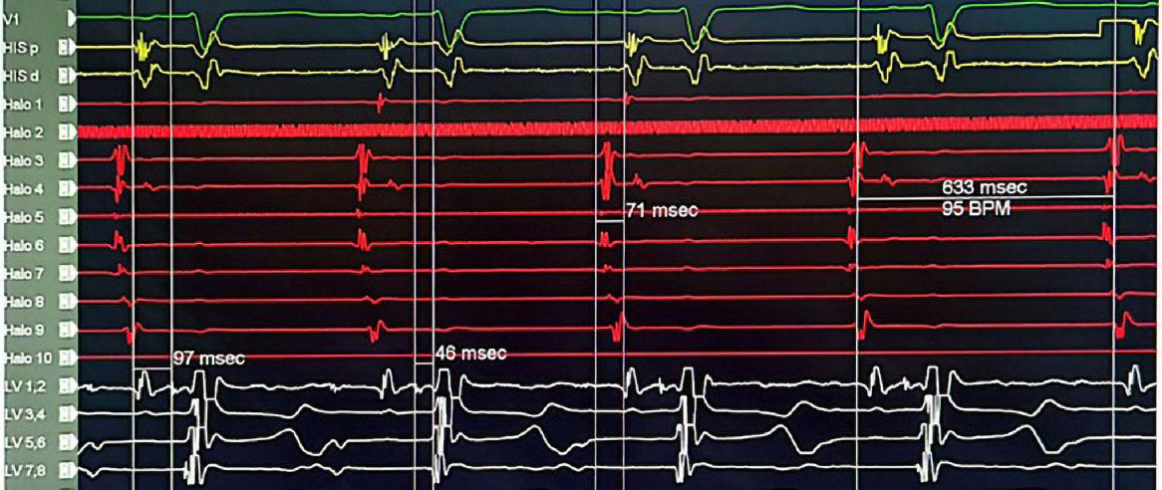
Figure illustrating the intracardiac intervals: A prolonged P-A interval of 71 ms was observed, with normal A-H (97 ms) and H-V (48 ms) intervals. The His catheter is positioned at the tricuspid annulus, the Halo catheter is placed in the right atrium, and the LV decapolar catheter is positioned on the left side of the AV septum.

His sinus intra-cardiac intervals: P-A = ms, A-H = 97ms, H-V = 48ms, with a sinus cycle length = 633 ms (*Figure 1*) while normal intervals are PA 25–55 ms (atrial to His bundle in the His bundle recording), AH 55–125 ms (AV node to His bundle)and HV 35–55 ms (His bundle to ventricle). Programmed atrial stimulation from Halo and CS catheters showed no ventricular pre-excitation and A-H jump. Programmed ventricular stimulation from the decapolar catheter in LVOT showed VA dissociation.

**Figure 2:**
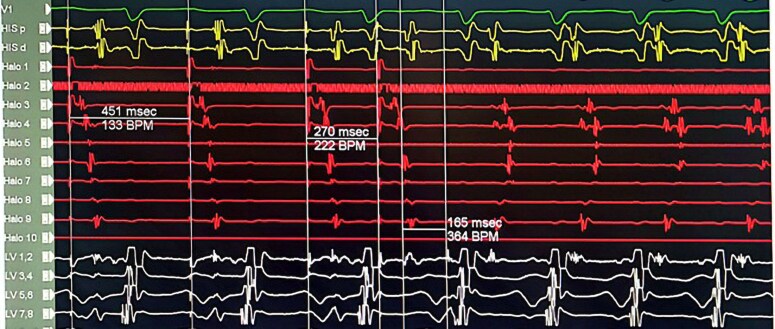
Programmed atrial stimulation during isoproterenol infusion induced the clinical tachycardia. A decrement in S1-S2 from 280 to 270 ms initiated the tachycardia with no A-H jump. The earliest atrial activation was located at the CS ostium area. The His catheter is positioned at the tricuspid annulus, the Halo catheter is placed in the right atrium, and the LV decapolar catheter is positioned on the left side of the AV septum.

**Figure 3:**
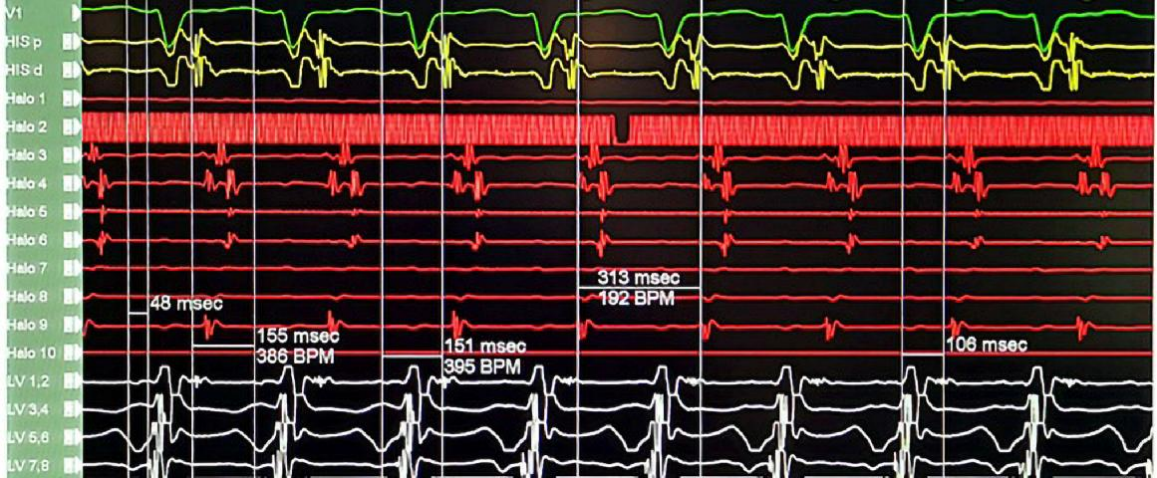
The intra-cardiac intervals during the tachycardia were as follows: A-H 155 ms, H-A 151 ms, septal VA 106 ms, H-V 48 ms, and tachycardia cycle length 313 ms. The His catheter is positioned at the tricuspid annulus, the Halo catheter is placed in the right atrium, and the LV decapolar catheter is positioned on the left side of the AV septum.

**Figure 4:**
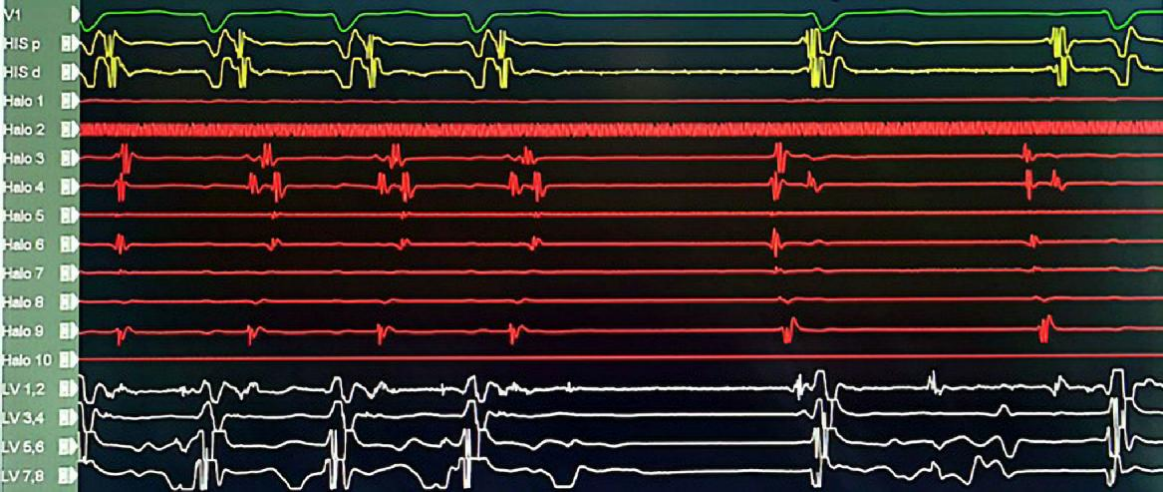
The induced tachycardia was non-sustained and terminated spontaneously with a P wave (atrial EGM on the Halo catheter).

Programmed atrial stimulation during isoproterenol infusion induced the clinical tachycardia. As shown in *Figure 2*, a decrement in S1-S2 from 280 to 270 ms initiated the tachycardia with no A-H jump. The earliest atrial activation was at the CS ostium. The intra-cardiac intervals during the tachycardia: A-H = 155ms, H-A = 151ms, septal VA = 106ms, H-V = 48ms and TCL = 313ms (*Figure 3*). The first initiating tachycardia beat had a VA interval similar to subsequent tachycardia beats, confirming the presence of VA linking. Atrial overdrive pacing at different rates during tachycardia demonstrated VA linking, thus excluding presence of atrial tachycardia. His synchronized PVC (premature ventricular contraction) did not reset the atrial EGM. This goes in favour of a node-dependent tachycardia while if such a PVC resets the tachycardia, there is likely an accessory pathway associated which is not in our case. The induced tachycardia was non-sustained and terminated spontaneously with a P wave (*Figure 4*). Diagnosis of atypical variant of AVNRT was made (*Table 1*).

**Table 1:** Key findings of EP study of patients which favoured AVNRT.

Characteristic	AVNRT
Effect of incremental atrial pacing	No pre-excitation seen in our patient.
AH jump	AH jump was not observed in our patient. It can be atypical AVNRT
VA linking	VA linking is seen.
Effect of HIS synchronous PVC	HIS synchronous PVC: did not reset the tachycardia in our patient. It favours AVNRT
Tachycardia termination with p wave was seen in our patient which is suggestive of node dependent tachycardia and makes AT unlikely	----

Given the uncertainty surrounding the compact AV node anatomy in post-AP Fontan patients, we chose to use the 3D electroanatomic mapping system (CARTO/Biosense Webster, USA). A virtual anatomic geometry of the right atrium was constructed by sequential point-to-point mapping with a Thermocool Smarttouch™ Catheter. Mapping was performed during sinus rhythm, as it was difficult to reinduce the tachycardia. The CS ostium and probable tricuspid annular position were mapped (see Supplementary material online, *Figure S4*). The anatomy of the triangle of Koch was challenging to define due to the inability to locate the AV node. Instead, the likely AV node area was tagged using the decapolar catheter from the LVOT. The slow pathway area was identified, and 4 cycles of radiofrequency(RF) lesions were applied, limited to 30–40 s, 55°C and 40 W of power. No junctional beats occurred despite consolidation of the RF lesions. The tachycardia was non-inducible by programmed electrical stimulation with isoproterenol. On serial follow-up at 1 week, 8 weeks, and 16 weeks, the patient remained asymptomatic, with no further tachycardia episodes recorded on an external loop recorder.

## Discussion

Performing AVNRT ablation in post-Fontan patients involves distinct challenges due to the complex anatomy, altered hemodynamics, and unique electrophysiological characteristics. While the procedure is typically successful, these patients face an elevated risk of complications and arrhythmia recurrence compared to the general population. A multidisciplinary team, including a paediatric interventional cardiologist, electrophysiologist, cardiothoracic surgeon, cardiac anaesthesiologist and a radiologist, is crucial. They ensure optimal access planning (transhepatic/surgical/hybrid), prepare for complications, facilitate real-time problem-solving (e.g. mapping through tortuous baffles or anomalous veins), and improve patient outcomes through collective expertise.

3D mapping systems help in anatomical reconstruction of distorted atria including baffles, tunnels, and surgical patches. They minimize radiation exposure. Activation and voltage mapping help to distinguish scarred tissue from viable myocardium.

IART is the most common arrhythmia in post-Fontan patients resulting from atrial dilation, surgical scars, and fibrosis, which create reentrant circuits within the RA.^1,2^ The use of a duo-decapolar catheter is instrumental in delineating the anatomy of RA and differentiating tachyarrhythmia.

CS Cannulation is challenging in post-Fontan patients, primarily due to altered RA anatomy. Factors like elevated RA pressure, venous congestion, or a persistent left SVC can contribute to CS dilatation.

In tricuspid atresia, there is absence or complete closure of the atrioventricular (AV) orifice of the morphological RV. Muscular atresia is the most common form in which there is a solid muscular floor to the atrium with absent tricuspid valve tissue.^3^ In our procedure, right heart catheterization and placement of a quadripolar catheter enabled us to accurately identify the anatomy of the tricuspid orifice.

The typical landmarks defining the Triangle of Koch may be altered in such patients. Dickinson *et al*. highlighted the abnormal positioning of the AV node in classical tricuspid atresia patients, noting that the RA aspect of the AV node was associated with the right atrioventricular sulcus, extending anteriorly beyond the insertion of the tendon of Todaro into the central fibrous body.^4^ In our case, despite extensive mapping of the right atrial septum, His potentials were located only after inserting a decapolar catheter into the LVOT. In tricuspid atresia, the AV node can usually be seen as a dimple in the RA floor, which connects directly to the left ventricle (LV).^4^ Hence, His bundle electrograms (EGMs) are easily identified from the left side of the septum.^5^

Although the PR interval was normal, intra-atrial conduction time was prolonged. Atrial dilation or displacement of the AV node may increase the inter-nodal distance, while conduction pathway disease can also contribute to delayed intra-atrial conduction.^5^

A diagnosis of the fast-slow variant of AVNRT was confirmed based on the following observations: (i) concentric atrial activation sequence with the earliest activation occurring at the CS-ostium; (ii) septal VA interval >70 ms; (iii) VA linking; (iv) VA dissociation during ventricular pacing; and (v) absence of atrial resetting with a His-synchronized PVC. Despite administering isoproterenol, an A-H jump was not observed. We were unable to perform ventricular overdrive pacing as the tachycardia was non-sustained and difficult to re-induce. No junctional beats were seen during slow pathway ablation.

AVNRT ablation in post-Fontan patient is challenging as (i) there is altered cardiac anatomy and physiology like lack of subpulmonay ventricle; AV node and HIS bundle may be displaced, malformed, or difficult to localize. (ii) Prior surgical baffles, patches, or tunnels create anatomical distortion and may cause fibrosis/scar tissue that complicates mapping and catheter manipulation. (iii) Anomalous conduction pathways or multiple atrial chambers may be present.

## Conclusion

AVNRT ablation in post-Fontan patients demands a tailored approach due to their unique anatomy and altered conduction pathways. This case highlights the pivotal role of advanced 3D mapping systems and a multidisciplinary team in overcoming procedural challenges.

## Lead author biography


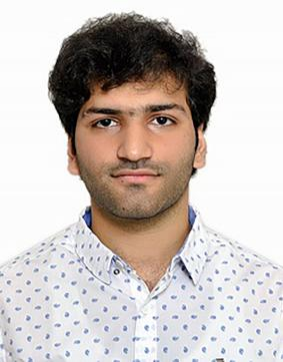


Dr. Atul Kaushik is a DrNB cardiology resident at Fortis Escorts Heart Institute, New Delhi, India. He has received accolades for paper and poster presentations at various national conferences. He has a keen interest in interventional cardiology and electrophysiology. He has 17 publications as corresponding author and/or first author under his name to date. He is an integral part of various other researches which are under process or under publication.

## Supplementary Material

ytaf569_Supplementary_Data

## Data Availability

The data underlying this article are available in the article.

## References

[1] Law IH, Alam O, Bove EL, Ohye RG, Bradley DJ, Yu S, et al Follow-up of a prospective surgical strategy to prevent intra-atrial reentrant tachycardia after the Fontan operation. Circ Arrhythm Electrophysiol 2016;9:e004478. 27979912 10.1161/CIRCEP.116.004478 PMC5166610

[2] Gelatt M, Hamilton RM, McCrindle BW, Gow RM, Williams WG, Trusler GA, et al Risk factors for atrial tachyarrhythmias after the Fontan operation. J Am Coll Cardiol 1994;24:1735–1741. 7963122 10.1016/0735-1097(94)90181-3

[3] Weinberg PM . Anatomy of Tricuspid atresia and its relevance to current forms of surgical therapy. Ann Thorac Surg 1980;29:306–311. 7362321 10.1016/s0003-4975(10)61476-2

[4] Dickinson DF, Wilkinson JL, Smith A, Becker AE, Anderson RH. Atrioventricular conduction tissues in univentricular hearts of left ventricular type with absent right atrioventricular connection (‘tricuspid atresia’). Heart 1979;42:1–8. 10.1136/hrt.42.1.1 PMC482104 475926

[5] Serratto M, Pahlajani DB. Electrophysiologic studies in tricuspid atresia. Am J Cardiol 1978;42:983–986. 727148 10.1016/0002-9149(78)90685-9

