# Supplementary material for: Challenges in AVNRT ablation in a patient with previous atrio-pulmonary Fontan surgery: a case report
Source: Eur Heart J Case Rep. 2025 Nov 7;9(12):ytaf569. doi: 10.1093/ehjcr/ytaf569 (PMC12693551; doi:10.1093/ehjcr/ytaf569)
Supplement: ytaf569_Supplementary_Data [file ytaf569_supplementary_data.docx]

**CHALLENGES IN AVNRT ABLATION IN A PATIENT WITH PREVIOUS ATRIO-PULMONARY FONTAN SURGERY: A CASE REPORT.**


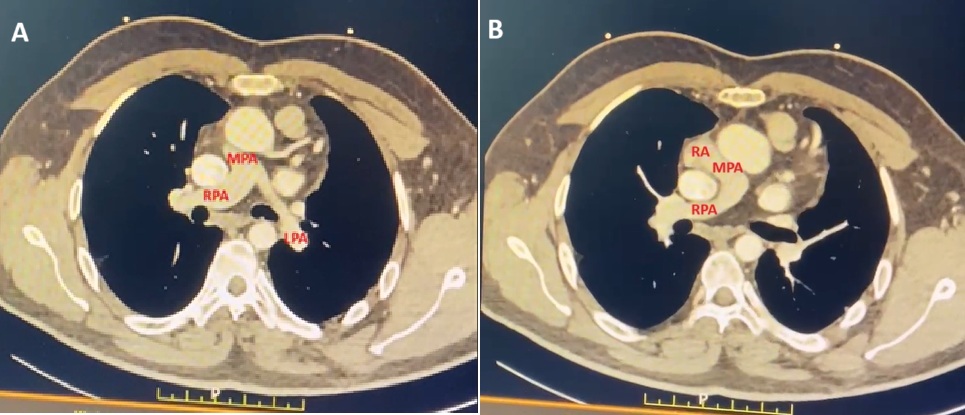


**Supplemenatry Figure 1**: CECT of the patient shows the intra-cardiac Fontan with main pulmonary artery (MPA) arising from the right atrium (RA) and divides into right pulmonary artery (RPA) and left pulmonary artery (LPA).


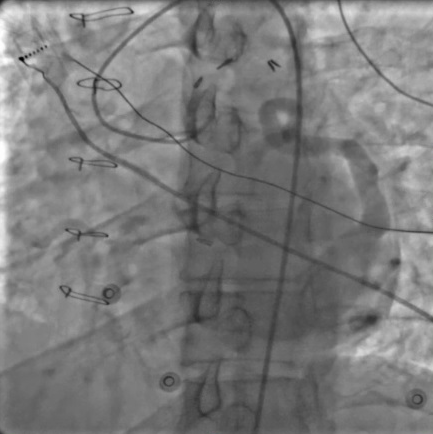


**Supplemenatry Figure 2**: Levophase coronary venography showing a grossly dilated coronary sinus.


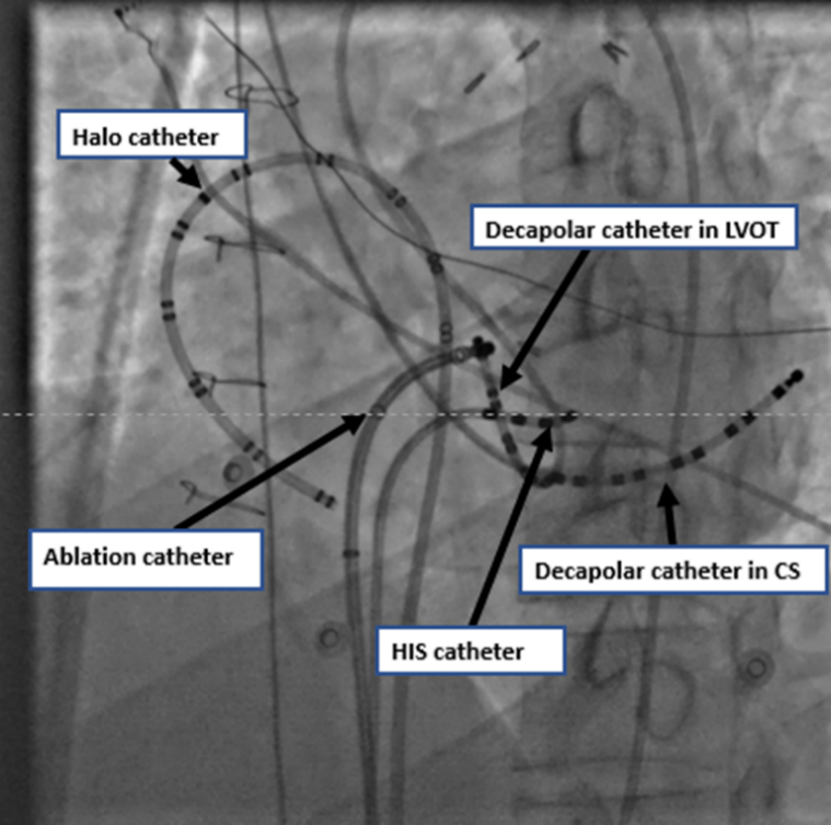


**Supplemenatry Figure 3:** Figure demonstrating the positioning of intracardiac catheters: The Halo catheter is positioned in the right atrium. A non-deflectable decapolar catheter is advanced through the internal jugular vein and positioned in the coronary sinus. The His catheter is utilized to locate the AV node but could not identify it, ultimately being positioned at the AV annulus. A deflectable decapolar catheter is inserted via the retrograde aortic approach to localize the AV node from the left side of the AV septum. The ablation catheter is positioned at the anticipated AV node location as identified from the left side.


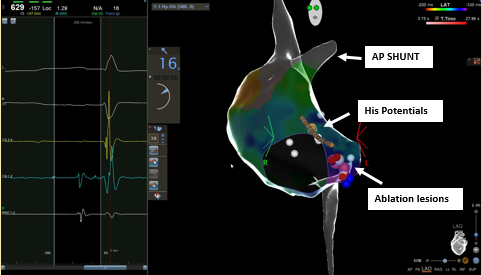


**Supplemenatry Figure 4**: A virtual anatomic geometry of the right atrium was constructed by sequential point-to-point mapping with a Thermocool Smarttouch™ Catheter. Mapping was performed during sinus rhythm. The CS ostium and probable tricuspid annular position were mapped. The likely AV node area was tagged using the decapolar catheter from the LVOT.
